# Intraspecific competition modulates heatwave effects on male reproduction in *Drosophila prolongata*

**DOI:** 10.1007/s00442-026-06006-0

**Published:** 2026-10-27

**Authors:** Shivanshi Gupta, Gayatri Raina, Marta Cortés Mediavilla, Tom Ratz

**Affiliations:** Department of Evolutionary Biology and Environmental Science, University of Zurich, Zurich, Switzerland

**Keywords:** Climate change, Extreme heat, Fruit fly, Global change, Heatwave, Intraspecific competition, Reproduction, Social interactions

## Abstract

Heatwaves are increasing in intensity, frequency, and duration and may drive the responses of organisms to climate change more strongly than gradual increases in mean temperature. The effects of heatwave events on wildlife remain poorly understood because the magnitude of these effects often depends on environmental the context: additional environmental stressors can exacerbate the impact of extreme temperatures. Here, we test whether the impact of extreme heat on adult reproduction and survival depends on the intensity of intraspecific competition, a key environmental variable determining access to resources, in male *Drosophila prolongata*, a species with extended male–male contests. Contrary to expectations, more intense intraspecific competition partially mitigated the negative effects of the heatwave on male reproductive output. This pattern may reflect a compensatory response whereby males increase reproductive investment under stronger competition, but only when this competition occurs in combination with the additional stress imposed by a heatwave. Overall, our findings reveal that interactions between heat stress and intraspecific competition can shape the impact of heatwaves on reproduction, and highlight the need to consider such interactive effects when evaluating the risk that extreme climatic events pose to wildlife.

**Supplementary Information:**

The online version contains supplementary material available at https://doi.org/10.1007/s00442-026-06006-0.

## Introduction

Exposure of wildlife to heatwaves, which are projected to become three- to six-fold more frequent in the coming decades (IPCC 2021), represents a major threat to biodiversity (Jørgensen et al. 2022; Ruthrof et al. 2018; Smale et al. 2019). The thermal stress associated with heatwaves can rapidly elevate mortality, disrupt somatic maintenance, and severely impair reproduction (Parratt et al. 2021; van Heerwaarden and Sgrò 2021; Walsh et al. 2019). Increasing evidence suggests that the effects of heatwaves on survival and reproduction are context-dependent and may be buffered or exacerbated by variation in key environmental factors such as humidity and water availability (Li et al. 2024; Padda et al. 2021), food availability (Stahlschmidt and Glass 2020), and exposure to pollutants (Tasmin et al. 2014). Biotic interactions can likewise mediate the impact of heatwaves, either mitigating or amplifying their detrimental effects (Stoks et al. 2017; Harvey et al. 2020). For example, the magnitude of heatwave effects on survival and reproduction may vary with predation risk (Stahlschmidt et al. 2024), pathogen exposure (Hector et al. 2021), and interspecific competition (Gao et al. 2024). Agonistic interactions among competing individuals are another important source of stress (MacLeod et al. 2023) that may alter responses to adverse conditions, such as heat stress. Yet relatively little attention has been given to the potential interaction between heat stress and intraspecific competition (Peñuelas et al. 2013). As a result, it remains unclear whether, and to what extent, the effects of heatwaves are mitigated or amplified by variation in the intensity of intraspecific competition.

Competition among conspecifics is a ubiquitous determinant of survival and reproductive success that can influence tolerance to heat stress (e.g. Mallard et al. 2020; Huxley et al. 2022). Increasing competition intensity may negatively affect individual condition by suppressing growth, immunity, and longevity (e.g. Graves and Mueller 1993; Hawley et al. 2006; Amundsen et al. 2007), thereby potentially reducing the ability of individuals to cope with heat stress. This is supported by studies showing that a higher density or greater proportion of competing individuals can reduce population growth or individual reproductive performance (e.g. Pandey et al. 2025; Porwal et al. 2025; De Nardo et al. 2025b). In turn, thermal stress associated with heatwaves may itself alter the nature and intensity of intraspecific competition (Amarasekare and Coutinho 2014), as suggested by previous studies showing that elevated temperatures can modulate competitive dynamics (García-Roa et al. 2020; Leith et al. 2021, 2022). For example, elevated temperatures exacerbate the negative effects of intraspecific competition on larval development in mosquitoes (Ezeakacha and Yee 2019) and on population growth in water fleas (Stockseth et al. 2025). Despite these insights, our understanding of the interactive effects of thermal stress and intraspecific competition remains limited (Leith et al. 2022). In particular, it is unclear whether the interactions observed under prolonged periods of warming (e.g., Laws and Belovsky 2010; Stockseth et al. 2025) also occur during short-term extreme events such as heatwaves. Understanding how these factors interact is essential for predicting how populations will cope with the compounding stressors expected under climate change (Chakraborty et al. 2025).

Here, we address this gap by investigating the interactive effects of heatwaves and intraspecific competition in the highly territorial fruit fly *Drosophila prolongata*. This species is ideal to explore this question because males compete physically in aggressive contests to gain access to females and food resources (Kudo et al. 2015). During these contests, males lunge, wrestle, and box using their elongated forelegs as weapons (Kudo et al. 2015), with larger and better-armed males gaining a competitive advantage (Amino and Matsuo 2020; Kikodze and Ratz 2025). *D. prolongata* inhabits cool, montane habitats in Southeast Asia (Singh and Gupta 1977; Toda 1991) and is particularly sensitive to heat: larvae fail to develop at temperatures exceeding 25 °C, and adults approach their upper thermal limits at 31 °C (Hitoshi et al. 2016; De Nardo et al. 2025b; A. Meena, *personal communication*). Growing evidence indicates that heatwaves reduce fertility and negatively affect reproductive traits in insects (e.g. Canal Domenech and Fricke 2023; Grandela et al. 2024; Cole et al. 2025; Morosinotto et al. 2025), suggesting that sublethal effects might be widespread yet often overlooked relative to more obvious lethal impacts. It remains, however, unknown whether the thermal stress caused by heatwaves can impair reproductive traits in *D. prolongata*. Moreover, although population density and male–male competition are expected to influence individual survival and reproductive success (Travis et al. 2023), it is currently unclear whether the effects of intraspecific competition are amplified or mitigated under heatwave conditions.

We used a factorial design to investigate the combined effects of heat stress and competition intensity on male reproductive output and survival. We exposed experimental males to either a control or an elevated temperature for five consecutive days during sexual maturation, a duration commonly used in heatwave studies (Meehl and Tebaldi 2004). We manipulated competition intensity during this period through variation in group size. We then quantified the number of offspring produced by each male following a single mating bout, subsequent survival, and testis size in a subset of males from each treatment group. If heatwaves and intraspecific competition act synergistically, for example if increasing competition intensity constrains thermal range limits (Mallard et al. 2020; Huxley et al. 2022), we would expect the negative effects of the heatwave to be exacerbated under more intense male–male competition. Specifically, males housed in larger groups should experience a greater reduction in reproductive output and survival under elevated temperature than males housed in smaller groups. Conversely, if heatwaves and intraspecific competition interact antagonistically, for example if males shift energy allocation towards current reproduction as a form of terminal investment (Williams 1966; Clutton-Brock 1984), we expect that the detrimental effects of heatwaves on reproduction will be attenuated under stronger male–male competition, whereas their negative effects on survival will be amplified. Under this scenario, males in larger groups would show a *smaller* reduction in reproductive output at elevated temperatures than males in smaller groups, reflecting increased investment in current reproduction. In contrast, males in larger groups are expected to exhibit a *greater* reduction in survival as a consequence of terminal investment.

## Materials and methods

### Origin and rearing of experimental flies

Experimental flies originated from wild *Drosophila prolongata* collected in October 2017 in Sa Pa, Vietnam, and were maintained as a large, outbred population for several generations under laboratory conditions (19 °C, 60% relative humidity, 14:10 h light: dark photoperiod) with ad libitum access to standard fly food. In the wild, the species occurs in a cool, humid mountain climate, with an average monthly temperature ranging from 10.1 °C to 20.1 °C (https://en.climate-data.org/), but temperatures can periodically exceed 30 °C. For example, daily maximum temperatures reached 36–38 °C in Sa Pa between 18 and 21 April 2023 (https://www.historique-meteo.net/). To produce experimental males, unmated males and females were collected within 24 h of eclosion and kept separately by sex until sexual maturation, which occurs approximately five days after eclosion (Matsuo 2018). At this stage, 100 males and 100 females were placed into a large cage (30 × 19 × 11 cm) and provided with Petri dishes containing an egg-laying substrate consisting of an agar-based jelly prepared with orange juice and coated with yeast paste to stimulate oviposition. Eggs collected from these dishes were transferred to 300-ml bottles containing standard fly food at a standardized density of 100 eggs per bottle. Bottles were maintained under the same temperature and humidity conditions as described above until adult emergence.

### Experimental design

Our aim was to test whether any detrimental effect of a heatwave on male reproductive output and survival depended on the intensity of male–male competition. To this end, we employed a fully factorial design in which groups of male *Drosophila prolongata* were exposed to one of two competitive regimes (small or large group size) and one of two heatwave treatments (control or heatwave). For practical reasons, we conducted the experiment in three separate blocks.

Newly eclosed males (0–2 days old) were randomly assigned to a treatment group and placed with unrelated males in 60-ml vials containing 12 ml of standard fly food. Males of *D. prolongata* reach sexual maturity several days after eclosion, with mating activity increasing over the first five days (Matsuo 2018; A. Meena, *personal communication*). The low-competition regime (small group) consisted of three males per vial, whereas the high-competition regime (large group) consisted of 10 males per vial. These group sizes were chosen based on prior work reporting stronger selection for competitive ability among males maintained at higher population densities (De Nardo et al. 2025a). Once groups were established, vials were immediately placed into incubators to initiate the thermal treatments. Relative humidity was monitored using a hygrometer (TA140 KlimaLogger, ATP Messtechnik GmbH, Germany) and maintained at approximately 50% for all treatment groups. Control males were maintained at 19 °C throughout the experiment. Males assigned to the heatwave treatment were exposed to a daytime temperature of 28 °C (8:00–20:00) for five consecutive days and a nighttime temperature of 19 °C. This duration is consistent with commonly used definitions of heatwaves (Meehl and Tebaldi 2004; Mu et al. 2025), which typically involve at least two consecutive days of abnormally high temperatures (Perkins and Alexander 2013; Crespi et al. 2020).

We chose 28 °C because preliminary tests conducted on a subset of males from the same laboratory population indicated that this was the highest temperature at which adult males can be maintained for five days without substantial mortality (Table S1). Although this temperature is well above the average temperatures likely experienced by the species in its natural habitat (10–20 °C), it falls within the range recorded during heatwaves across its native range. For example, in the region from which our population originates daily maximum temperatures in October reached or exceeded 28 °C on eight occasions lasting at least three consecutive days between 2010 and 2019 (mean duration = 7 days, range = 3–15 days; https://www.historique-meteo.net/). We selected a five-day exposure because it matches the duration of male sexual maturation (Matsuo 2018) and remain short relative to the adult lifespan (30–42 days; Kudo 2021; Macartney and Bonduriansky 2022) and ecologically relevant for this species.

### Mating assays, longevity, and testis size

On the fifth day of exposure to the competitive and thermal treatments, all vials were transferred to a temperature-controlled room at 19 °C to initiate mating assays. Each experimental male was placed individually into a 60-ml vial containing a sexually mature, unmated female and a small dish lined with yeast paste as an oviposition substrate. Females originated from the same outbred population and had been housed in groups of up to 20 individuals in 60-ml vials containing 12 ml of standard food. They were maintained at 19 °C under control conditions for at least five days prior to the assay to ensure sexual maturation. Males were removed after 24 h of mating. Females were allowed to oviposit for an additional 24 h, after which vials containing oviposition substrate were frozen for subsequent counting of unhatched eggs and first-instar larvae. All measurements were conducted blind to treatment where possible. In total, we measured the reproductive output of 278 males: *n* = 33 males at 19 °C in small groups, *n* = 33 males at 28 °C in small groups, *n* = 104 males at 19 °C in large groups, and *n* = 108 males at 28 °C in large groups.

Following the mating assay, males were transferred to individual 60 ml vials containing agar media (to prevent desiccation) but no food to assess survival under starvation. Resistance to starvation, commonly used as a proxy for adult longevity (e.g. De Nardo et al. 2025b; Meena et al. 2024a), was recorded as the number of days from the end of mating until death. Vials were checked twice daily (morning and afternoon) to determine survival status.

Lastly, we aimed to determine whether any detrimental effects of the experimental treatments on reproduction were associated with altered testis development during sexual maturation. Although the precise timing of testis development has not been described in *D. prolongata*, it is likely similar to that of other *Drosophila* species, in which the testes develop largely during the pupal stage and continue maturing during early adulthood (Fabian and Brill 2012). Males achieve higher courtship and mating success approximately five days after eclosion, presumably reflecting the completion of sexual maturation (Matsuo 2018). We therefore measured testis size as a proxy for testis development and investment in sperm production (Schärer et al. 2008) by freezing a subset of experimental males five days after the experimental treatment began. These males were then dissected in a droplet of phosphate-buffered saline under a stereomicroscope. Testes were extracted, transferred to a droplet of paraffin oil to facilitate straightening, and photographed using an sCMOS Microscope camera K5 mounted on a Leica M165 FC stereomicroscope (Leica Microsystems GmbH, Wetzlar, Germany) at 32× magnification and 144 pixels per inch. Testis surface area was measured from photographs using Fiji (version 2.14.0). For each male, the surface area of both testes was measured and averaged to obtain mean surface area, which provides an indication of sperm number and male investment in sperm production (Pitnick 1996; Markow and O’Grady 2008). We excluded images in which both testes were damaged during dissection. When only one testis was damaged, we used measurements from the intact testis. This resulted in a total of 98 males included in subsequent analyses: *n* = 13 males at 19 °C in small groups, *n* = 11 males at 28 °C in small groups, *n* = 37 males at 19 °C in large groups, and *n* = 37 males at 28 °C in large groups.

### Statistical analysis

All statistical analyses were conducted in R version 4.4.3 (R Development Core Team 2024) using the packages *car* (Fox et al. 2016), *MASS* (Ripley et al. 2017), *aod* (Lesnoff and Lancelot 2015), *lme4* (Bates et al. 2014), *lmerTest* (Kuznetsova et al. 2017), *glmmTMB* (Brooks et al. 2017), and *emmeans* (Lenth 2025). Model assumptions were assessed using diagnostic plots to evaluate normality of residuals and homoscedasticity. Models fitted with Poisson and binomial error distribution were also examined for overdispersion. All models included temperature treatment (control 19 °C versus heatwave 28 °C), group size (3 versus 10 males), their interaction, and block number (1–3) as fixed effects, and vial ID as a random effect. Block number was included as a fixed effect rather than a random effect because it comprised only three levels (Harrison 2015). The number of unhatched eggs and the number of larvae were modelled using zero-inflated negative binomial distributions. Total offspring number and resistance to starvation were modelled using a Conway–Maxwell–Poisson (COM-Poisson) distribution to account for overdispersion (Shmueli et al. 2005; Lynch et al. 2014). We did not use a Cox proportional hazards model to analyse resistance to starvation because lifespan was recorded for all individuals, such that no observations were censored. Moreover, the data violated the proportional hazards assumption of the Cox model (Therneau 2015; χ² = 12.3, *P* = 0.006). Hatching success and the proportion of hatched eggs were analysed using a binomial error distribution. Testis size was analysed using a Gaussian error distribution. Whenever the heat × group size interaction had a significant effect, we further conducted post hoc comparisons among treatment combinations using estimated marginal means (EMMs) derived from the fitted model, with pairwise contrasts adjusted for multiple testing using the Bonferroni method.

## Results

### Reproductive output and resistance to starvation

**Fig. 1:**
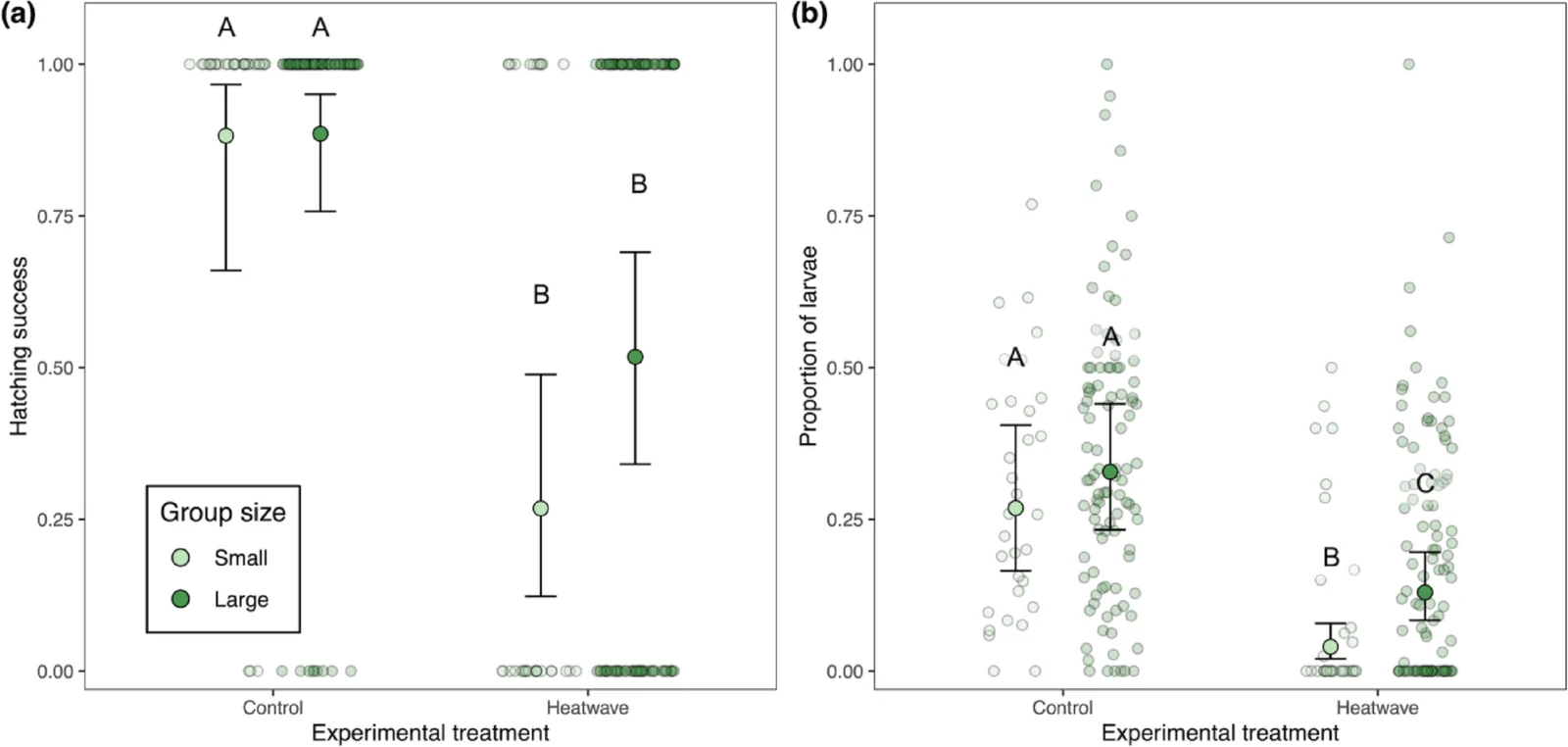
Effects of the heatwave treatment (control or heatwave) and group size (small or large) on hatching success (**a**) and the proportion of hatched eggs 48 h following mating (**b**). Dots with error bars represent model-predicted means ± 95% confidence intervals. Circles in dull colour represent raw data points

Overall, 97.8% of matings between experimental males and stock females resulted in offspring production. There was no significant difference in the number of unhatched eggs or the total number of offspring produced (unhatched eggs and larvae combined) between treatment groups (Table 1). However, treatments differed in hatching success. Specifically, eggs sired by males exposed to a heatwave showed a 39.4% reduction in hatching success compared to eggs sired by control males. This was supported by a significant negative main effect of the heat treatment, with no significant effect of group size and no heat × group size interaction on hatching success (Table 1; Fig. 1a). As a consequence, the number of larvae was lower for heatwave males (Table 1; log-scale contrast = 0.317 ± 0.144 SE, z = 2.19, *P* = 0.028). However, this reduction in the number of larvae produced depended on group size, as indicated by a significant heat × group size interaction on the proportion of eggs hatching into larvae (Table 1; Fig. 1b). The negative effects of heat exposure on larval production were weaker in males maintained in larger groups compared to those kept in smaller groups (Table 2).

**Table 1:** Effects of the interaction between the heatwave treatment (control or heatwave) and group size (small or large) and the main effects of heatwave treatment and group size on male reproductive output. Values are obtained from GLMMs

	Number of unhatched eggs			Number of larvae			Total offspring			Hatching success			Proportion of larvae		
	LRχ^2^	df	*P*	LRχ^2^	df	*P*	LRχ^2^	df	*P*	LRχ^2^	df	*P*	LRχ^2^	df	*P*
Heatwave treatment (control/heatwave)	0.009	1	0.921	6.16	1	**0.013**	3.10	1	0.078	17.7	1	**< 0.0001**	21.9	1	**< 0.0001**
Group size (small /large)	3.07	1	0.079	0.286	1	0.592	2.84	1	0.091	0.003	1	0.955	0.797	1	0.371
Heatwave treatment: Group size	0.684	1	0.408	2.74	1	0.097	1.56	1	0.211	1.36	1	0.242	4.18	1	**0.040**
Block number	0.693	2	0.707	6.95	2	**0.030**	0.561	2	0.453	1.16	2	0.280	1.28	2	0.256

LR = likelihood ratio. Statistically significant *P* values (< 0.05) are shown in boldface

**Table 2:** Pairwise comparisons between treatment groups for the proportion of larvae sired by experimental males. Values were obtained using estimated marginal means and *P* values adjusted using the Bonferroni correction

	Proportion of larvae			
	Estimate	SE	Z	*P*
Control–Small vs. Heatwave–Small	2.18	0.424	5.14	**< 0.0001**
Control–Small vs. Control–Large	−0.282	0.315	−0.893	0.808
Control–Small vs. Heatwave–Large	0.899	0.321	2.80	**0.025**
Heatwave–Small vs. Control–Large	−2.46	0.375	−6.56	**< 0.0001**
Heatwave–Small vs. Heatwave–Large	−1.28	0.374	−3.42	**0.0034**
Control–Large vs. Heatwave–Large	1.18	0.252	4.68	**< 0.0001**

Statistically significant *P* values (< 0.05) are shown in boldface

**Fig. 2:**
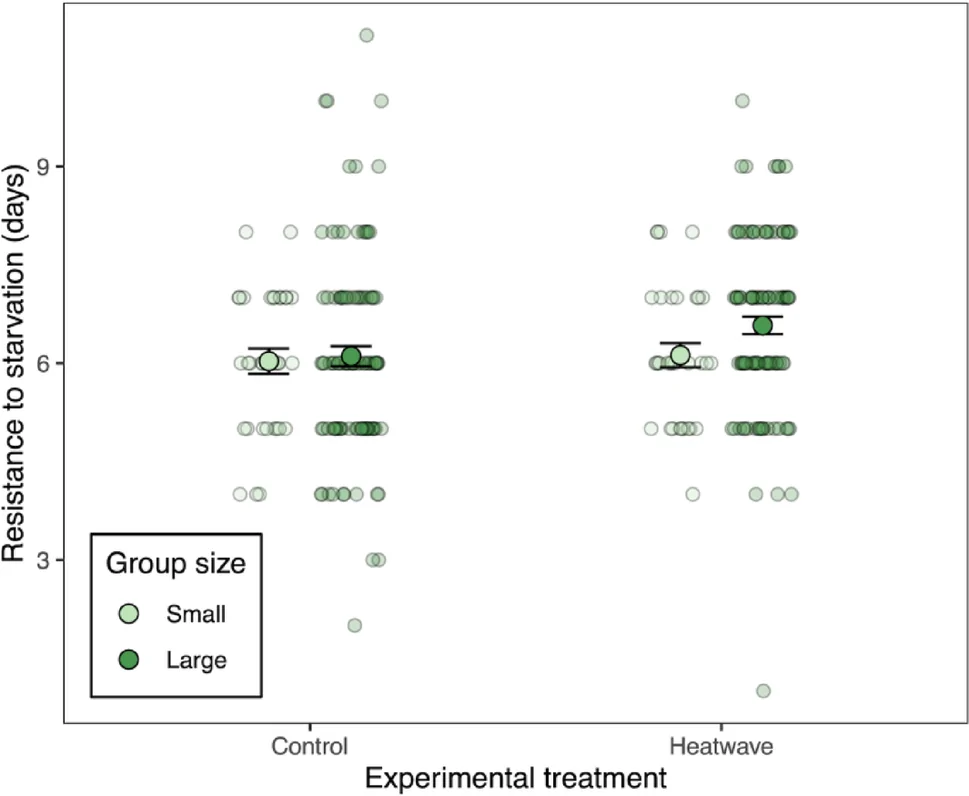
Effects of the heatwave treatment (control or heatwave) and group size (small or large) on male resistance to starvation. Dots with error bars represent means ± standard errors. Circles in dull colour represent raw data points

Males exposed to the heatwave showed no significant difference in resistance to starvation (Table 3; Fig. 2) compared to control males. Group size and the heat × group size interaction had no significant effects on starvation resistance (Table 3). Finally, there was a significant positive effect of block number, indicating that males in later blocks survived longer under starvation conditions (Figure S1).

**Table 3:** Effects of the interaction between the heatwave treatment (control or heatwave) and group size (small or large) and the main effects of heatwave treatment and group size on male survival and testis size. Values are obtained from GLMMs

	Male lifespan			Testis size		
	LRχ^2^	df	*P*	LRχ^2^	df	*P*
Heatwave treatment (control/heatwave)	0.084	1	0.771	0.988	1	0.320
Group size (small /large)	0.086	1	0.769	0.252	1	0.615
Heatwave treatment: Group size	0.973	1	0.323	0.015	1	0.902
Block number	52.8	2	**< 0.0001**	0.072	2	0.788

LR = likelihood ratio. Statistically significant *P* values (< 0.05) are shown in boldface

### Testis size

Testis size was not affected by the heat treatment, group size, their interaction, or block number (Table 3). These findings suggest that the effects of the heatwave treatment and group size during sexual maturation on male reproductive output were unlikely to be mediated by differences in overall testis development.

## Discussion

We investigated the combined effects of a heatwave and competition intensity on male reproductive output and survival in the territorial fruit fly *Drosophila prolongata*. Our first main finding was that competition among males kept in larger groups partially mitigated the negative effects of heat stress on larval production. This suggests that males may increase their investment in reproduction when facing more intense competition (Parker et al. 1996; Kokko et al. 2012; Lüpold et al. 2020), but only when this competition is combined with the additional stress imposed by a heatwave. However, this compensatory response was only partial: males experiencing stronger competition still produced fewer larvae than males maintained at the control temperature (19 °C). These results indicate that the effects of heatwaves on individual reproduction may be more complex than traditionally recognized, echoing recent work reporting environmental dependence of heatwave effects at the population level (e.g. De Nardo et al. 2025b; Pandey et al. 2025). Identifying the mechanisms underlying the interaction observed here and in other studies should therefore be a priority. Mechanisms that modulate the magnitude of heat stress effects could have important implications for understanding the impact of extreme climatic events on wildlife. For example, if populations at lower densities are more vulnerable to heatwaves, drivers of population decline such as habitat fragmentation or overexploitation could interact synergistically with heat stress, accelerating population extinction relative to the effects of each factor in isolation. Overall, our results suggest that reduced reproductive output following heatwaves may arise both from direct physiological effects of heat stress and from indirect effects mediated by changes in population density or competition intensity.

The positive effects of group size on reproductive output under a heatwave likely reflect a response to increased competition among males. Males experiencing more intense competition may respond by investing more resources in reproduction to secure greater reproductive success. Such increases in reproductive investment have been documented in several *Drosophila* species, where males adjust their mating behaviour and ejaculate allocation in response to anticipated sperm competition. For example, studies in which focal males were housed with one to five rivals for a period comparable to that used here (5 days) generally report longer mating durations than in males housed alone (e.g. Filice et al. 2020; Lizé et al. 2012; Mazzi et al. 2009; Price et al. 2012). Likewise, males exposed to a greater risk of sperm competition, owing to the presence of rivals before and/or during mating, often increase sperm production and transfer during mating (e.g. Garbaczewska et al. 2013; Moatt et al. 2014; Price et al. 2012; Wigby et al. 2009). There is also evidence that such adjustments in reproductive investment in response to increased competition among males can lead to higher reproductive output (Price et al. 2012), as observed in our study. Regardless of the specific mechanisms underlying heat–competition interactions, such surprising responses may be more common than expected. Indeed, recent evidence from burying beetles shows that following a heatwave males achieve greater reproductive success under intense male competition than under lower levels of competition (Sidhu et al. 2026). Overall, these findings, together with those reported here, indicate that responses to an increased risk of mating or sperm competition can moderate the effects of heatwaves.

Such association between male reproductive allocation and population density has been widely documented across taxa (reviewed in Simmons and Fitzpatrick 2012 and Lüpold et al. 2020). For example, males reared at high larval density invest more in testes and sperm production in the Indian meal moth *Plodia interpunctella* (Gage 1995), produce larger spermatophores in the armyworm *Pseudaletia separata* (He and Tsubaki 1992), and developed larger testes in the dung fly *Scatophaga stercoraria* (Stockley and Seal 2001) and the leech *Helobdella papillornata* (Tan et al. 2004). Male house mice invest more in ejaculate production when they perceive more rival males in their environment (Ramm and Stockley 2009). Sperm morphology also changes in response to variation in the intensity of intrasexual competition in Gouldian finches (Immler et al. 2010) and anole lizards (Kustra et al. 2019). Our findings add to this body of evidence by showing that the effects of adult male density and male reproductive output in *Drosophila prolongata* may depend on environmental context. Specifically, we found that group size affected male reproductive output only in heatwave-exposed males and not in control males. This pattern may reflect that males maintained at control temperatures invest close to their reproductive maximum regardless of their perceived risk of sperm competition. By contrast, compensating for the detrimental effects of heatwave exposure on reproductive function may be costly, such that only males facing stronger competition are willing to make this investment to secure at least some reproductive success.

The overall detrimental effect of heat stress on reproductive output was expected and aligns with the growing number of studies documenting negative impacts of heatwaves on the reproductive function in ectotherms (e.g. Grieve and Pilakouta 2026; Meena et al. 2024a; 2024b; Pilakouta et al. 2024; Sales et al. 2018, 2021), including cases where relatively mild heatwaves have non-lethal effects (Parratt et al. 2021; Ratz et al. 2024; van Heerwaarden and Sgrò 2021; Walsh et al. 2019). Our results resonate with recent work on the interplay between intraspecific competition and responses to heat stress in *Drosophila*, including our study species, showing negative consequences of short-term (≤ 12 h) heat stress during larval development on adult body size, male fertility, and sperm competitive ability (De Nardo et al. 2025b; A. Meena, *personal communication*). Together, these findings provide clear evidence that heat stress can impair the reproductive function of male *D. prolongata*. Notably, given the affinity of this species for cooler climates, an ambient temperature of only 28 °C during sexual maturation was sufficient to significantly reduce male reproductive success. Interestingly, previous work on this species has reported stronger negative effects of heat stress on sperm competitiveness in populations that evolved under more intense male–male competition (De Nardo et al. 2025b), suggesting that populations experiencing stronger intrasexual competition may evolve increased vulnerability to heat stress. At the population level, such effects could increase the risk of population decline or extinction under recurrent heatwaves in populations experiencing stronger sexual selection (Pandey et al. 2025). However, whether this prediction applies broadly across species is unclear. Interactions between elevated temperatures and sexual selection can have no effect on individual reproduction and survival (Porwal et al. 2025), and in some cases could even enhance population fitness (Parrett and Knell 2018; Martínez-Ruiz and Knell 2017).

We also found that temperature and group size affected neither male lifespan nor testis size, whereas lifespan was significantly influenced by block number. Specifically, resistance to starvation, our surrogate measure for male lifespan, increased across successive blocks, suggesting that males in later blocks were able to survive longer without food. Although this pattern may simply reflect stochastic variation among blocks, it is also possible that males emerging later were on average in better condition. Importantly, because males were haphazardly assigned to treatments within each block and treatments were balanced across blocks, any variation in male condition among blocks cannot have biased our estimates of the effects of temperature and group size. More generally, this result highlights the importance of accounting statistically for potential block effects, even in experiments conducted under highly standardized laboratory conditions. Meanwhile, the fact that male survival was affected by neither the heatwave treatment nor competitive environment is surprising because greater investment in reproduction should be expected to come at a cost. In *D. melanogaster*, while males sometimes suffer a reduction in survival as a result of increased ejaculate allocation under greater risk of sperm competition (e.g., Bretman et al. 2013; Guo and Dukas 2020), the opposite pattern has been reported (e.g., Moatt et al. 2013). Interestingly, recent evidence suggests that the lifespan reduction sometimes observed is not caused by greater mating investment, but could instead stem from direct effects of interacting with rivals (Smithson et al. 2024). Additional research is needed to identify the proximate mechanisms underlying the lasting costs of social interactions with rivals.

The absence of differences in testis size is surprising given that male reproductive success was influenced by the combined effects of temperature and group size. Although heat stress during larval development can impair spermatogenesis and increase the risk of cytological abnormalities and spermatid mortality (Canal Domenech and Fricke 2022, 2023), these mechanisms are unlikely to explain the interactive effect observed here. Instead, reproductive success may have been mediated by other processes, such as variation in the quantity or quality of sperm transferred by males (Snook 2005; McGraw et al. 2007), the composition of seminal fluid proteins (Simmons and Fitzpatrick 2012), accessory gland maturation (Bangham et al. 2002; Ruhmann et al. 2016), or female sperm ejection (Manier et al. 2010). Although it remains unknown whether thermal stress or male competition during sexual maturation influences any of these processes, previous work in *D. prolongata* suggests that male siring success can vary through plastic adjustments of sperm transfer (De Nardo et al. 2021). Beyond sperm transfer, further research is needed to document the timing of gonad development and the production of sperm and seminal fluid proteins in *D. prolongata*. Unlike *D. melanogaster*, in which males emerge with mature sperm and can sometimes mate within hours of eclosion (Pitnick et al. 1995; Ruhmann et al. 2016), *D. prolongata* males do not fully exhibit mating behaviour until approximately five days after eclosion (Matsuo 2018). It is therefore assumed that sexual maturation in this species, including the completion of sperm development, continues for several days after adult emergence. The production of viable and competitive sperm may require prolonged developmental processes, such as sperm elongation, which can greatly delay male sexual maturation (Pitnick et al. 1995). Clarifying whether such processes are sensitive to heat stress and male competition will require further study of the physiological mechanisms underlying male reproductive development.

Overall, our findings, together with previous work, highlight the importance of considering intraspecific competition, such as group size or population density, when assessing the effects of climatic conditions like heatwaves. By revealing how interactions within groups can modify the detrimental effects of heat stress on reproduction, our results have broader implications for conservation initiatives and for future research aimed at mitigating the impact of climate change. If the pattern observed here proves to be general, animal populations at higher densities or with more limited access to resources may appear surprisingly less affected by environmental stress simply because individuals increase their reproductive investment under stronger competition. At the same time, our results suggest that the effects of intraspecific competition on male reproduction may sometimes become evident only under stressful conditions, such as thermal stress. This idea is supported by previous studies showing stronger fitness consequences of intraspecific competition when populations are exposed to other stressors, including predators (Richarson and Ingram 2025), limited food resources (Turner 2004), pesticides (Op de Beeck et al. 2018), or inbreeding (Meagher et al. 2000). Taken together, these findings provide further support for the view that the outcome of intraspecific interactions is strongly dependent on environmental context (Chase et al. 2002; Chamberlain et al. 2014; Fisher et al. 2021).

In conclusion, the magnitude of heatwave effects may often depend on other ecological variables, such as the intensity of competition within a population or group of interacting individuals. In our study, we found evidence that more intense male–male competition can *reduce* the magnitude of the negative effects of a heatwave on male reproduction. Heatwaves, which are increasing in intensity, frequency, and duration (Perkins et al. 2012; Perkins-Kirkpatrick and Lewis 2020), are expected to drive the responses of organims to climate change more strongly than gradual increases in mean temperature (Easterling et al. 2000; Ma et al. 2021; Sentis et al. 2013). To fully understand the impact of climate change on biodiversity there is therefore a pressing need to move beyond the traditional critical thermal limit framework and investigate how heat stress affects fertility (Walsh et al. 2019). Our findings contribute to this effort by showing that the extent to which heatwaves affect reproduction can be mediated by environmental conditions. We encourage future work to examine how the effects of heatwaves vary with other environmental variables, such as resource abundance, pathogen exposure, or predation risk (Hector et al. 2021; Sentis et al. 2013; Walberg 2024). Such studies will be crucial in advancing our understanding of how heatwaves, and extreme climatic events more generally, affect wildlife under biologically realistic conditions.

## Supplementary Information

Below is the link to the electronic supplementary material.

Supplementary Material 1

## Data Availability

Data and analysis code are available from the OSF repository https://osf.io/4x6ha/overview?view_only=fcdd80e9da364c039da2cca5cb9e24b7.
