# Supplementary material for: Intraspecific competition modulates heatwave effects on male reproduction in Drosophila prolongata
Source: Oecologia. 2026 Oct 27;208(11):162. doi: 10.1007/s00442-026-06006-0 (PMC13714135; doi:10.1007/s00442-026-06006-0)
Supplement: Supplementary file 1 [file 442_2026_6006_MOESM1_ESM.docx]

**Electronic supplementary material**

**Intraspecific competition modulates heatwave effects on male reproduction in *Drosophila prolongata***

**Table S1:** Mortality of male flies following 5-day temperature exposure. Two replicate vials housing 16 to 30 adult males were exposed to each of the three temperatures (28.0, 29.0, and 30.5°C) for five consecutive days.

| Temperature |  | Replicate # |  | Individuals | Dead | Mortality |
| --- | --- | --- | --- | --- | --- | --- |
| 28.0°C |  | 1 |  | 16 | 0 | 0.0% |
| 28.0°C |  | 2 |  | 28 | 0 | 0.0% |
| 29.0°C |  | 1 |  | 18 | 2 | 11.1% |
| 29.0°C |  | 2 |  | 26 | 7 | 26.9% |
| 30.5°C |  | 1 |  | 18 | 18 | 100.0% |
| 30.5°C |  | 2 |  | 30 | 30 | 100.0% |

**Figure S1.** Effect of block number on male resistance to starvation. Dots with error bars represent means ± standard errors. Circles in dull colour represent raw data points.
